# A scoping review on the relationship between race/ethnicity and the receipt of supportive care medications during cancer treatment: Implications for the clinical pharmacist

**DOI:** 10.1002/jac5.1727

**Published:** 2022-11-13

**Authors:** MegCholack Awunti, David L. DeRemer, Sherise Rogers, Lisa Scarton, Lauren Adkins, Diana J. WIlkie, John M. Allen

**Affiliations:** ^1^ Department of Pharmacotherapy and Translational Research University of Florida College of Pharmacy Orlando Florida USA; ^2^ Department of Pharmacotherapy and Translational Research University of Florida College of Pharmacy Gainesville Florida USA; ^3^ Department of Family, Community, and Health Systems Science University of Florida College of Nursing Gainesville Florida USA; ^4^ University of Florida Health Science Center Libraries Gainesville Florida USA; ^5^ Department of Biobehavioral Nursing Science University of Florida College of Nursing Gainesville Florida USA

**Keywords:** cancer, chemotherapy‐induced nausea and vomiting, clinical pharmacist, health disparities, pain management, palliative care, supportive care

## Abstract

There is surmounting levels of evidence on the health disparities within cancer treatment in the United States (US). Most of the research focused on cancer specific factors including anticancer incidence, screening, treatment and follow‐up, and clinical outcomes such as overall survival (OS). Less is known about the disparities present with supportive care medication use in cancer patients. Supportive care utilization during cancer treatment has been linked to improved quality of life (QoL) and OS among patients. The goal of this scoping review is to summarize findings of current literature on the relationship between race and ethnicity and the receipt of supportive care medications during cancer treatment for pain and chemotherapy‐induced nausea and vomiting (CINV). This scoping review was conducted following the Preferred Reporting Items for Systematic Reviews and Meta‐Analysis (PRISMA‐ScR) guidelines. Our literature search included quantitative studies, qualitative studies, and grey literature written in the English language with clinically relevant outcomes pertaining to pain and CINV management in cancer treatment published from 2001 to 2021. Articles that met the predefined inclusion criteria were considered for inclusion in the analysis. The initial search yielded 308 studies. Following deduplication and screening, 14 studies met the predefined inclusion criteria, with majority of the studies being quantitative studies (n = 13). Collectively, results were mixed results regarding the presence of racial disparities for supportive care medication use. Half of the studies (n = 7) supported this finding whereas, the other half (n = 7) did not identify any racial disparities. In our review, multiple studies illustrate the existence of disparities in the use of supportive care medications in some cancer types. Clinical pharmacists should strive to eliminate supportive medication use disparities as part of a multidisciplinary team. In order to develop strategies to prevent supportive care medication use disparities in this population, further research and analysis of external factors that influence them are needed.

## 1 INTRODUCTION

Cancer survivorship is an aspect of cancer care focused on alleviating the challenges patients encounter during and after a cancer diagnosis, including patients on maintenance treatment.^1^ Cancer survivorship addresses the coordination of clinical care, provision of financial and psychosocial support, and assistance with reintegration into society.^1^ A critical component of cancer survivorship involves managing symptom burden with the use of supportive care, as this has a profound impact on health‐related quality of life (HR‐QoL). Cancer patients experience many symptoms that are both treatment and disease induced. Common symptoms encountered by patients include nausea and vomiting, fatigue, pain, insomnia, and depression. These adverse events could worsen treatment response and negatively impact HR‐QoL, increase treatment interruptions, and shorten OS. Several investigations have demonstrated that early integration of supportive cancer care prolongs OS.^2^, ^3^, ^4^ In a study by Basch et al, improvements in patient‐reported outcomes such as HR‐QoL and mood were associated with improved OS.^5^ The authors hypothesized that survival benefits might be associated with early symptom control.^5^ Furthermore, effective use of supportive care has been associated with other positive benefits, such as decreased utilization of healthcare resources.^6^ These findings support current cancer treatment guidelines, which recommend early integration of supportive care services into a patient's treatment plan after diagnosis.^7^, ^8^

The Multinational Association of Supportive Care in Cancer (MASCC) defines supportive care in cancer as “the prevention and management of the adverse effects of cancer and its treatment”.^8^ With the development of novel therapeutics and technologies, the integration of supportive care across the cancer continuum continues to expand. Owing to their importance in optimizing care, numerous high‐quality clinical practice guidelines have emerged for various supportive care topics.^9^, ^10^, ^11^, ^12^, ^13^ Embedded in these guidelines are evidence‐based clinical recommendations demonstrating the impact of supportive care interventions, including supportive care medications (SCM). Much of the evidence on the importance of SCM in cancer is focused on the effective management of chemotherapy‐induced nausea and vomiting (CINV) and cancer‐related pain. Early and optimized use of SCM is crucial to achieve symptom control, improve HR‐QOL and enhance treatment‐related outcomes.

Cancer health disparities in historically minoritized patients have been well‐described for several facets of care, including supportive care management. Much of the previous research has focused on disparities regarding the utilization of palliative care services and referrals to supportive care centers. Cole et al conducted a retrospective cohort study comparing the receipt of palliative care services among patients with metastatic cancer in minority‐serving hospitals vs nonminority‐serving hospitals, with patients receiving care at minority‐serving hospitals had 33% lower odds of receiving palliative care, as compared to nonminority serving hospitals (OR 0.67 [0.53‐0.84]). When accounting for race and ethnicity, Asian patients had lower odds of receiving palliative services compared to White patients (OR 0.93 [0.88‐0.98]), while Hispanic patients had 6% higher odds of receipt of care than White patients (OR 1.06 [1.01‐1.10]). No differences were found among Black patients when compared to White patients.^14^ Additionally, Ju et al assessed palliative care (PC) utilization rates among patients with metastatic foregut cancers (MFC) and factors associated with PC receipt. When assessing by race and ethnicity, minority patients (Black [OR 0.87 (0.84‐0.91)], Hispanic [OR 0.72 (0.68‐0.76)], and Asian [0.85 (0.79‐0.91)] patients) had lower odds overall for receiving palliative care. ^15^ Regarding early referral to palliative care to achieve symptom control, White patients are more likely to receive an early referral for severe pain [OR 2.86 (1.88‐4.35)] and depressed mood [OR 3.45 (1.71‐6.99)] compared to minority patients.^16^ Disparities in the quality of supportive care provided to historically minoritized patients has also been described with common themes of increased need for self‐advocacy and inadequate patient education on symptom burden and treatment side effects.^17^ These findings indicate that palliative care in historically minoritized cancer patients is commonly overlooked and can have negative consequences.

However, despite our understanding of disparities in palliative cancer, there is limited evidence describing racial disparities in using SCM in cancer management. Identifying and mitigating any potential disparities in the use of SCM is crucial to promoting improved symptom control and HR‐QoL for all patients. The objective of this scoping review is to^1^ summarize the findings on racial and ethnic disparities in the use of SCM in cancer patients, with a particular focus on pain and CINV^2^; identify current gaps with our understanding of disparities in the use of SCM^3^; describe the vital role of the clinical pharmacist on the multidisciplinary team to alleviate this disparity.

## 2 MATERIALS AND METHODS

This scoping review study was conducted following the Preferred Reporting Items for Systematic reviews and Meta‐Analyses extension for Scoping Reviews (PRISMA‐ScR) reporting guidelines.^18^ A health sciences librarian (L.E.A.) developed the search strategy using the specified inclusion and exclusion criteria. Searching was conducted in May 2022 through the databases PubMed, EMBASE, and SCOPUS. The search included relevant subject headings, truncation, and phrase‐searching in the title and abstract fields on the research topics of racial and ethnic disparities, supportive care medication, cancer treatment, pain, and chemotherapy‐induced nausea and vomiting. Search limits were restricting publications to the English language and the publication years of 2001 to December 2021. The studies included were retrospective cohort studies and observational studies. This study included two main types of materials, namely peer‐reviewed published literature and grey literature. An example set of search terms are detailed in Table S1.

Studies were included in our analysis if they met all the following criteria^1^: evaluation of SCM use among patients receiving treatment for cancer by race and ethnicity; and^2^ assessment of cancer‐related pain and/or CINV management. Studies were excluded from our analysis for any of the following reasons^1^: lack of evaluation of SCM used for the relief of cancer‐related pain or CINV^2^; lack of comparison by race and ethnicity.

### 2.1 Data extraction, synthesis of results, and quality assessment

All potential studies were independently evaluated by two reviewers. In the event of disagreements between the two initial reviewers, J.A. served as the third reviewer. Title and abstract screening were initially carried out to identify empirical literature sources. Next, empirical articles underwent a full‐text review, and articles that met the inclusion criteria proceeded to the data extraction phase. Extraction forms were designed to collect pertinent variables, including patient demographics, location, study design, cancer type, and study outcomes of pain and CINV management. Data extraction was carried out independently by two reviewers and then compared routinely by both reviewers to ensure consistency with the collection and presentation of information. To assess the risk of bias and quality of included articles, the ROBINS‐E and ROBINS‐I tools were utilized according to study design.^19^

## 3 RESULTS

**FIGURE 1:**
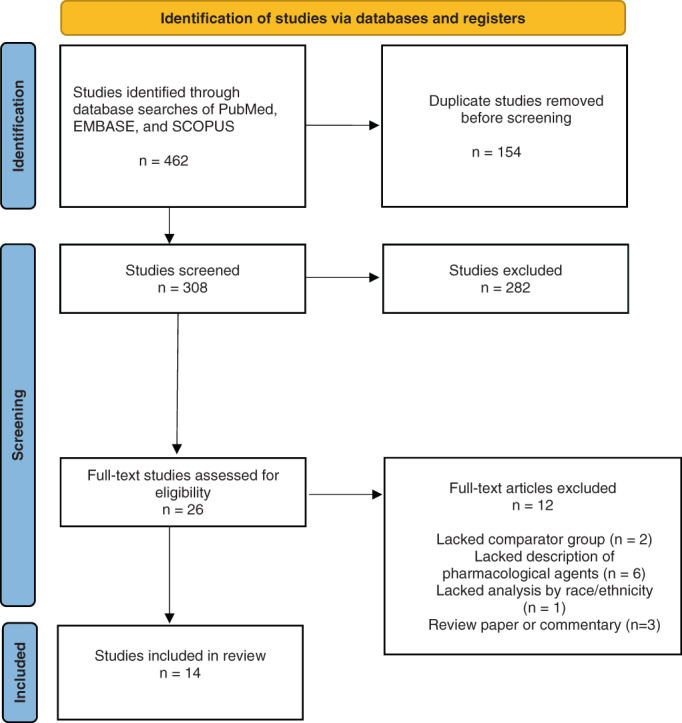
Study flow diagram

After our initial database search, a total of 308 studies were identified. After deduplication, title, and abstract screening, 14 studies met inclusion criteria and were included in the final analysis (Figure 1). Of the 14 included studies, 11 were available as full‐text articles, and 3 were only available in abstract form. Regarding reported study outcomes, there were 13 quantitative studies and 1 qualitative study. Among the included quantitative studies, 11 were retrospective cohort studies utilizing datasets or patient records, including the SEER‐Medicare database (n = 7), Texas cancer registry‐Medicare linked database (n = 1), Navigating cancer database (n = 1), and the National‐level cancer registry and linked health records (n = 1). Two studies utilized chart reviews, and one study was based on data derived from a prospective observational trial. Thirteen studies took place in the United States, and one study was performed in New Zealand. A full description of included studies is provided in Table 1.

**TABLE 1:** Description of included studies

Reference (n)	Study design and data source	Location	Cancer type	Variable assessed (pain, ^a^CINV, or both)	Participants	Major study findings	Covariates
Wieder et al, 2014 (n = 360)	Retrospective cohort study (quantitative)	United States	Multiple	Pain	12.5% White 47.5% Black 5% Asian 33.3% Hispanic 1.7% not specified	When predicating long‐acting opiate use by race/ethnicity, no differences were identified between other racial groups and Black patients Asian or Hispanic ^b^OR 0.606, *P* = 0.158 White ^b^OR 1.007, *P* = 0.987	Adjusted for confounders
Pinheiro et al, 2019 (n = 23 091)	Retrospective cohort study (quantitative) ^c^SEER‐Medicare linked database	United States	Breast cancer	Pain	88.2% US‐born non‐Hispanic 4.1% US‐born Hispanic 5.6% Foreign‐born non‐Hispanic 2.1% Foreign‐born Hispanic	In terms of opioid use, foreign‐born NH and foreign‐born Hispanics were less likely to utilize opioid medications compared to US‐born NH. US‐born Hispanics were more likely to utilize opioids compared to US‐born NH. US‐born Hispanics ^d^RR *1.06 (1.02‐1.10), P < 0.01* Foreign‐born non‐Hispanics ^d^RR *0.91 (0.87‐0.95), P < 0.001* Foreign‐born Hispanics ^d^RR *0.86 (0.80‐0.93), P < 0.001*	Adjusted for socio‐economic co‐variates (marital status, urban/rural region, SEER geographic region, census tract income quartile, dual eligible, and low‐income subsidy)
Lu et al, 2022 (n = 10 745)	Retrospective cohort study (quantitative) ^c^SEER‐Medicare linked database	United States	Pancreatic cancer (adenocarcinoma)	Pain	75.3% White 8.8% Black 6.9% Asian 8.6% Hispanic Other/unknown	Among patients with pancreatic cancer, opioid initiation did not vary by race/ethnicity Black ^d^RR 0.99 (0.96‐1.02), *P* = 0.44 Asian ^d^RR 1.00 (0.97‐1.03), *P* = 0.88 Hispanic ^d^RR 1.01 (0.98‐1.04), *P* = 0.48 Other/Unknown ^d^RR 1.01 (0.92‐1.11), *P* = 0.82	Adjusted for patients' socio‐demographics, tumor characteristics, and treatment
Lamba et al, 2020 (n = 17 957)	Retrospective cohort study (quantitative) ^c^SEER‐Medicare linked database	United States	Brain cancer (metastatic)	Both	75.2% White 11.4% Black 6.7% Asian 6.9% Hispanic	Regarding nonopioid analgesics, racial/ethnic disparities were not identified Black ^b^OR 0.89 [0.66‐1.20], *P* = 0.44 Hispanic ^b^OR 0.69 [0.46‐1.05], *P* = 0.08 Asian ^b^OR 0.97 [0.70‐1.34], *P* = 0.86 Asians had lower odds of utilizing opioid medications for pain management compared White patients Black ^b^OR 0.96 [0.85‐1.08], *P* = 0.48 Hispanic ^b^OR 1.09 [0.94‐1.26], *P* = 0.24 Asian ^b^ *OR 0.86 [0.75‐0.99], P = 0.04* Asian and African American patients had lower odds of using antiemetics for the management of CINV compared to White patients Black ^b^ *OR 0.75 [0.68‐0.83], P < 0.001* Hispanic ^b^OR 0.91 [0.80‐1.04], *P* = 0.16 Asian ^b^ *OR 0.83 [0.73‐0.94], P = 0.004*	Covariates were adjusted for age, sex, marital status, ZIP code level high school completion rate and median household income, year of BM diagnosis, Charlson comorbidity index, and primary tumor site
Hwang et al, 2004 (n = 89)	Retrospective cohort study (quantitative)	United States	Prostate cancer	Both	57.3% White 42.7% Black	Regarding opioids, NSAIDs, and antiemetic use within the last 6 months of life, medication utilization did not vary by race/ethnicity Opioids: *X* ^2^ = 0, *P* = 0.87 NSAIDs: *X* ^2^ = 0.87, *P* = 0.35 Antiemetics: *X* ^2^ = 1.27, *P* = 0.26	Not reported
Gurney et al, 2021 (n = 20 081)	Retrospective cohort study (Quantitative) New Zealand cancer registry and National Pharmaceutical data collection	New Zealand	Lung cancer	Pain	83% non‐Māori 20.2% Māori	In terms of medication access by drug class (nonopioids, mild/moderate opioids, and strong opioids) differences were not identified based on ethnicity. Minor differences were identified among specific medications. Māori patients had lower odds of receiving NSAIDs and codeine phosphate but higher odds of receiving tramadol compared to non‐Māori patients Accessing any pain medicine: ^b^OR 1 (0.85 to 1.19) Accessing nonopioids: Overall ^b^OR 0.89 (0.79 to 1.01) NSAIDs ^b^ *OR 0.73 (0.64‐0.83)* Accessing mild or moderate opioids: ^b^OR 1 (0.9 to 1.12) Accessing strong opioids: Overall ^b^OR 1.08 (0.96 to 1.23) Codeine Phosphate ^b^ *OR 0.88 (0.77‐0.99)* Tramadol ^b^ *OR 1.19 (1.02‐1.4)* Māori patients had higher odds of receiving strong opioids less than 2 weeks before death and lower odds of receiving strong opioid medication greater than 24 weeks prior to death comparative to non‐Māori patients. Timing to first medication access (Strong opioids) Before death: <2 weeks ^b^ *OR 1.3 (1.07‐1.59)* >24 weeks ^b^ *OR 0.66 (0.52‐0.83)*	Adjusted for age and sex
Gomez et al, 2012 (n = 4566)	Retrospective cohort study (quantitative) Texas Cancer Registry (TCR)‐Medicare linked database	United States	Lung cancer	^a^CINV	87% White 8.4% Black 3.2% Hispanic 1.4% other	Black patients were less likely to adhere to CINV prophylaxis treatment on highly or moderately emetogenic chemotherapy for lung cancer than White patients ^*b*^ *OR 0.672 (0.538‐0.839), P = 0.001*	Adjusted results for each significant variable in the logistic regression model
Check et al, 2016 (n = 883)	Quantitative ^c^SEER‐Medicare linked database	United States	Breast cancer	Pain	85% White 15% Black	Racial disparities were not identified with the use of opioids for end‐of‐life care among Black and White female patients with stage 4 breast cancer White vs Black ^d^RR 0.97 (0.84‐1.13)	Adjusted for clinical characteristics, namely age, year of cancer diagnosis, medical comorbidity, and receipt of cancer‐directed therapy
Check et al, 2016 (n = 1130)	Retrospective cohort study (quantitative) ^c^SEER‐Medicare linked database	United States	Breast cancer	^a^CINV	90% White 10% Black	Considering CINV‐related utilization, no racial and ethnic disparities were identified. Any ^d^RR 0.66 (0.42‐1.04) Outpatient visits ^d^RR 0.67 (0.43‐1.06) Any post‐chemotherapy utilization ^d^RR 0.95 (0.71‐1.03)	Adjusted for clinical characteristics (age at diagnosis, year of chemotherapy initiation, tumor characteristics, and medical comorbidity)
Check et al, 2016 (n = 1130)	Retrospective cohort study (quantitative) ^c^SEER‐Medicare linked database	United States	Breast cancer	^a^CINV	90% White 10% Black	For any NK1 use and aprepitant use, Black women were less likely to utilize these medications compared to White women on highly emetogenic chemotherapy Any NK1 use ^d^ *RR 0.68 (0.51‐0.91)* Aprepitant use ^d^ *RR 0.54 (0.35‐0.83)* Fosaprepitant use ^d^RR 0.82 (0.51‐1.33)	CINV (primary model) adjusted for patient age at diagnosis, year of chemotherapy initiation, and tumor characteristics
Booker et al, 2020 (n = 64)	Retrospective cohort study (qualitative) Secondary data analysis of a cluster randomized controlled trial	United States	Multiple	Pain	50% White 50% Black	In the hospice setting, no racial differences were identified with regarding medication orders for appropriate opioid and nonopioid medications in pain management *P* = 0.3173 Black patients in hospice were less likely to have a bowel regimen order (both existing and new) for opioid‐induced constipation compared to White patients *P = 0.0563*	Not reported
Smith et al, 2014 (n = 583)	Prospective observational study (quantitative)	United States	Not reported	Pain	72% non‐Minority 28% Minority	At baseline, minority patients were less likely to be taking pain medications (*P* < 0.001) or report relief from pain medications (*P* = 0.05)	Not reported
Osazuwa‐Peters et al, 2021 (n = 3762)	Retrospective cohort study (quantitative) Navigating cancer database	United States	Head and Neck Cancer	Pain	62.8% White 37.2% non‐White	White patients were more likely than non‐White patients to receive a new prescription for pain management •^*b*^ *OR =4.58; 95% CI 2.23‐9.38*	Adjusted for covariates, abstract
Dranitsaris et al, 2012 (n = 2640)	Retrospective cohort study (quantitative)	United States	Breast cancer (metastatic)	^a^CINV	75% White 25% Black	Antiemetic support was similar between White and Black patients White patients (77.3%) vs Black patients (78.8%), *P* = NS^e^	Not reported

^a^ CINV, chemotherapy‐induced nausea and vomiting.

^b^ RR, relative risk.

^c^ SEER, surveillance, epidemiology, and end results.

^d^ OR, odds ratio.

^e^ NS, not stated.

The ROBINS‐E tool was utilized for quantitative studies (n = 13), and the ROBINS‐I tool was utilized for qualitative studies (n = 1). Regarding the risk of bias for included studies, most studies were of moderate (n = 9) or high risk of bias (n = 4). The most reported potential source of bias was attributed to using datasets and patient records as the primary information source. Further information regarding risk of bias can be found in Table S2.

Considering the race and ethnicity of participants, six studies primarily focused on comparing outcomes in Black and White patients only, six studies evaluated disparities among multiple races, and two studies evaluated disparities based on ethnicity. Regarding cancer type, two studies evaluated multiple types of cancers, one study did not report the assessed cancer type, and 11 studies evaluated specific cancer types, including breast cancer (n = 5), lung cancer (n = 2), prostate cancer (n = 1), brain cancer (n = 1), pancreatic cancer (n = 1), and head and neck cancer (n = 1). Accounting for the primary objective, eight studies evaluated pain management, four studies assessed CINV management, and two studies evaluated variables associated with both pain and CINV management.

### 3.1 Supportive Care medication use and racial and ethnic disparities

### 3.2 Pain management

Variables associated with pain medication use were analyzed in eight studies. Two studies comprised solely of Black and White participants, four studies accounted for multiple races, and two compared pain management across ethnicities. Opioids were the most evaluated drug class for pain management among patients diagnosed with cancer. The results of the articles will be presented based on the racial and ethnic demographics of each study.

For studies focusing on Black‐White differences, two articles were evaluated. Check et al assessed supportive care medication use among Black and White female patients with stage IV breast cancer.^20^ No racial disparities were identified with the utilization of opioid medications for female patients with stage IV breast cancer. Conversely, Booker et al performed a secondary data analysis of a completed cluster randomized controlled trial that evaluated differences in nurse‐provided cancer pain management among Black and White patients in hospice.^21^ In the hospice setting, discrepancies were not identified regarding the appropriateness of opioid and nonopioid medications for pain management or with written pain management plans. However, racial disparities were identified with bowel regimens. Black patients were less likely to have both a new and existing bowel regimen order compared to White patients.

For studies assessing multiple races, four studies were evaluated. All the articles were quantitative studies. Two of the studies provided the racial and ethnic breakdown of participants, while the other two articles categorized participants by minority vs nonminority or White vs non‐White.

Lu et al conducted a population‐based retrospective cohort study utilizing the SEER‐Medicare database.^22^ The study evaluated opioid use in elderly patients with pancreatic cancer from 2007 to 2015 and revealed that race (Black [OR 0.99 (0.96‐1.02)], Asian [OR 1.00 (0.97‐1.03)], Hispanic [OR 1.01 (0.98‐1.04)]) as a variable did not drive the initiation of opioid medication among this patient group. Wieder et al determined the impact of prescription coverage on the initiation of long‐acting opioid medications among indigent minority populations via a retrospective chart review.^23^ The study participants comprised of Black patients (n = 171), Hispanic patients (n = 120), White patients (n = 45), and Asian patients (n = 13). Black patients were used as the reference group for the analysis due to the large sample size. A logistic regression analysis of long‐acting opiates use revealed no differences among Asian or Hispanic patients (OR 0.606, *P* = 0.158) and White patients (OR 1.007, *P* = 0.987) when compared to Black patients.^23^

Smith et al compared palliative care outcomes among minority patients (Black and Hispanic) and nonminority patients.^24^ Researchers found that minority patients were less likely to be on pain medications (*P* < 0.001) or experience symptomatic relief from pain medications (*P* = 0.05). Symptomatic improvements and disparities in medication use were alleviated with regard to pain management following palliative care consultations.^24^ Lastly, Osazuwa‐Peters et al evaluated the sociodemographic variables of patient‐reported pain outcomes among patients with head and neck cancer in the community setting.^25^ When assessing patterns associated with new prescription patterns for pain medications, it was found that White patients had higher odds of being prescribed new pain medications compared to non‐White patients [OR: 4.58 (2.23‐0.38)].

For studies based solely on ethnicity, two articles were identified. Gurney and colleagues aimed to describe access and timing to pain medications among Māori and non‐Māori patients with lung cancer in New Zealand.^26^ Data was derived from the New Zealand Cancer Registry and linked health records. Pertaining to access to pain medications, no disparities were identified among both ethnicities. Some differences were noticed with specific medications. Māori patients had lower odds of receiving NSAIDs (OR 0.73 [0.64‐0.83]) and codeine phosphates (OR 0.88 [0.77‐0.99)] compared non‐Māori patients. Māori patients also had 19% higher odds of receiving tramadol than non‐Māori patients (OR 1.19 [1.02‐1.4]). Regarding timing to access of pain medications, disparities were identified in correlation with the utilization of strong opioids. Compared to non‐Māori patients, Māori patients had 34% lower odds of receiving strong opioids greater than 24 weeks before death [0.66 (0.52‐0.83)] and 3% higher odds of receiving strong pain medications less than 2 weeks before death [1.3 (1.07‐1.59)]. Indicating that ethnicity played a role in access to strong pain medications for Māori patients. Similarly, a native study carried out by Pinheiro et al assessed differences in supportive medication use by ethnicity and nativity.^27^ The primary focus was on Hispanic/Latino patients. The study revealed that foreign‐born non‐Hispanics and foreign‐born Hispanics were 9% (RR 0.91 [0.87‐0.95]) and 14% (RR: 0.86 [0.80‐0.93]), respectively, less likely to utilize opioid medications than US‐born non‐Hispanics. Conversely, US‐born Hispanics were 6% more likely to utilize opioids than US‐born non‐Hispanics (RR: 1.06 [1.02‐1.10]), suggesting that cultural norms based on nationality can affect opioid utilization.

### 3.3 CINV management

Four studies exclusively evaluated CINV management. Among these, three articles evaluated Black‐White disparities. Check et al conducted two studies assessing antiemetic use in Black and White women with breast cancer on highly emetogenic chemotherapy. Both studies were retrospective cohorts utilizing the SEERs‐Medicare linked database. The first study evaluated racial disparities with neurokinin‐1‐receptor antagonists (NK1 RA) for CINV prophylaxis.^28^ It revealed that with regards to any NK1‐RA and aprepitant use, Black women were 32% (RR: 0.68 [0.51‐0.91]) and 46% (RR: 0.54 [0.35‐0.83]) respectively, less likely to use these medications when compared to White women.

The second study with the same investigators explored racial variations in antiemetic use and post‐chemotherapy healthcare utilization.^29^ No racial differences were identified with post‐chemotherapy utilization. Similar to the previous study, Dranitsaris et al did not identify any racial disparities between Black and White patients pertaining to antiemetic support.^30^

Gomez et al assessed adherence to National Comprehensive Cancer Network guidelines for antiemesis prophylaxis for patients with lung cancer undergoing chemotherapy.^31^ This was a retrospective cohort study with data from the Texas Cancer Registry‐Medicare linked database. The primary agents assessed were serotonin‐5‐HT3 receptor antagonists and dexamethasone. The study revealed that Black patients had 33% lower odds of being adherent to CINV prophylaxis on highly or moderately emetogenic chemotherapy compared to White patients (OR: 0.672 [0.538‐0.839]). No differences were identified among other racial groups and White patients.

### 3.4 Studies evaluating both pain and CINV management

Lambda and colleagues conducted a multiracial and ethnic analysis of supportive care medication use among patients diagnosed with brain metastasis.^32^ The study consisted of White, Black, Hispanics, and Asians. In terms of opioid use, Asians had 14% lower odds of using opioids for pain management compared to White patients (0.86 [0.75‐0.99]). No racial disparities were identified with African American and Hispanic patients when compared to White patients for opioid use. Additionally, racial differences were not identified with nonopioid analgesics. Regarding antiemetic agents, Black and Asian patients were 25% (0.75 [0.68‐0.83]) and 17% (0.83 [0.73‐0.94]) respectively less likely to utilize antiemetic medications compared to White patients.

The second study was carried out by Hwang et al^33^ The study primarily compared racial differences between White and African American patients with prostate cancer who received care through a Veterans Affairs clinical setting within the last 6 months of life. The study evaluated the utilization of both opioid and nonopioid analgesic agents. No racial disparities were identified with either medication class. When accounting for antiemetic support, racial disparities were not discovered as well.

## 4 DISCUSSION

The intent of this scoping review was to summarize the current evidence on the presence of SCM use disparities in patients with cancer, with a particular emphasis on pain and CINV management. Our review revealed that racial and ethnic disparities in the utilization of SCM have been reported across multiple cancer types. However, the results from the various studies included in our analysis were heterogeneous, with some studies identifying disparities^23^, ^26^, ^27^, ^28^, ^31^, ^32^ whereas others did not identify any disparities.^20^, ^21^, ^22^, ^29^, ^33^ Among the things we believe to have driven this finding are differences in how comparison groups were defined. For example, some studies found disparities with one minority group, while others found disparities among two or more minority groups. This suggests considerable differences among racial/ethnic minority groups and that grouping racial and ethnic minority patients into a catch‐all “minority” group classification may mask between‐group differences. In our study, nine of the studies made majority/minority comparisons (ie, Black/White), while five studies made multiracial comparisons. Multiple authors have advocated the need for a more disaggregated approach to exploring racial/ethnic health disparities,^34^, ^35^ as several ethnicities can exist within each racial group. It also beacons the need for increased consistency in how racial and ethnic minority patients are defined to enable the assessment of racial disparities and the creation of methods to alleviate them.

Beyond stratification by racial groups, we evaluated the occurrence of racial and ethnic disparities by supportive care treatment type. We utilized three treatment categories, including pain (n = 8), CINV (n = 4), and studies assessing both pain and CINV management (n = 2). Regarding pain management, 5 studies did not identify racial and ethnic disparities, while 3 identified disparities. The results from the other two categories were evenly distributed regarding racial and ethnic disparities. Half of the studies identified disparities, while the other half did not. We could not determine drivers for racial and ethnic disparities by supportive care treatment type, but we identified that most of the studies assessed pain medication use. These findings imply there is a need for more research exploring the social and clinical influences for racial and ethnic disparities with SCM use. As well as evaluating symptoms beyond pain.

This study has several strengths. Most studies utilized nationally recognized databases. These databases are representative of patients with cancer and provide confirmation that the observed patterns stem from real‐world occurrences. Furthermore, the study period spanned 20 years. The aim of this extensive study period was to ensure that all relevant literature within this research sector captured and identify trends over time. Only one study was carried out between 2001 and 2010.^33^ All other studies were carried out between 2010 and 2021, implying that this is an upcoming research area.

This scoping review is not without its limitations. First, among several studies, minority groups were underrepresented as compared to their proportion of the overall population.^21^, ^22^, ^28^, ^29^, ^31^, ^33^ In some studies, after adjusting for confounding factors such as sociodemographic and clinical factors, statistical significance was lost. Due to the small sample sizes among racial and ethnic minorities, the possibility of a type II error cannot be excluded. Among included studies, only one study had a sample size in which a minority patient group represented the largest number of participants.^23^ Future studies assessing SCM disparities should aim to reflect a racially diverse patient population and allow for more robust comparisons between and within minority patient groups.

Second, most of the studies were quantitative, primarily retrospective cohort analyses from large datasets. Inherent to this study design, the investigators were unable to extract clinical outcomes, patient preferences, or experiences regarding treatment.^20^, ^27^, ^28^, ^29^, ^31^, ^32^, ^33^ Significant gaps in the current literature exist. Based on current evidence, it is unclear the extent to which patients utilized the medications or received symptomatic relief. Additionally, studies highlighted that the cause of this disparity is multifactorial, specifically noting both clinical and social variables.^28^, ^29^, ^32^ Financial constraints and location of services were recurringly discussed as barriers to medication access among patients, particularly for marginalized patient populations. Some articles also discussed a provider‐level barrier associated with the inability of providers to recognize symptoms among racial/ethnic minorities.^27^, ^32^ Patient‐level barriers were mentioned as well, including limited expression of concerns to providers,^28^, ^29^, ^32^ cultural differences,^20^, ^27^, ^29^, ^32^ and language barriers.^27^, ^32^ However, these factors were not consistently included in study analyses. Only one study in this review carried out a qualitative analysis.^21^ The authors did not find differences in pain management, but racial disparities were identified regarding the management of opioid‐induced constipation.^21^ This raises the question about the presence of disparities in the management of side effects stemming from supportive care medication. Further exploration of the impact of social determinants of health on supportive care medication use disparities is needed.

Third, most studies focused on the Medicare patient population comprising solely of those 65 years and older.^20^, ^22^, ^27^, ^28^, ^29^, ^31^, ^32^ The findings produced from these studies may not be generalized to younger cancer patients and those with other types of insurance.

### 4.1 Role of the clinical pharmacist in addressing disparities with the use of SCM among minority patient populations

While the demand for oncology services is steadily increasing, there is a projected shortage of oncology practitioners to address the demand.^36^ Considering the cornerstone of supportive care and symptom management is pharmacotherapy, this provides an opportunity to elevate the pharmacist's role on the multidisciplinary team to further assist oncology practitioners to optimize patient health outcomes. Clinical pharmacists, including those with specialized training in oncology, are expertly trained to provide a plethora of services to cancer patients, including medication reconciliation, preparation and dispensing of chemotherapy, assisting in mitigating financial toxicity incurred by patients, monitoring drug‐drug and drug‐disease interactions, and providing patient education on medications.^37^, ^38^, ^39^, ^40^, ^41^, ^42^, ^43^, ^44^, ^45^, ^46^, ^47^ Several studies have shown that clinical pharmacist involvement in the cancer care process resulted in better patient satisfaction and enhanced control of side effect profiles overall.^40^, ^43^, ^44^, ^48^ These findings infer that pharmacists are positioned to help alleviate usage disparities with supportive care medications in patients with cancer.

Clinical pharmacists can provide patient education on chemotherapy and provide tools to enhance medication adherence. Patients are educated on complicated chemotherapy regimens, adverse events and mitigation strategies, and supportive care treatment options for symptom management.^37^, ^38^, ^40^, ^41^ Pharmacists can aid in the creation of patient‐tailored education tools and handouts to enhance understanding of the disease state and medications. Pharmacists engage in continuity of care communication with patients to assess medication efficacy, alleviate symptom burden, and address any other concerns from patients.

A patient‐centered treatment approach can be utilized by pharmacists to build trust with their patients. Pharmacists can provide one‐on‐one counseling sessions,^37^ giving patients ample time to express concerns about the medication regimens, side effect profiles, life concerns, and enable patient autonomy in the treatment process. This is particularly important as our aging population has increased more comorbidities and chronic disease medications that add to the complexity of care. Providing a safe space for patients to voice concerns, medication reconciliation, and side effect mitigation through counseling can positively impact health outcomes and patient satisfaction.^38^

**FIGURE 2:**
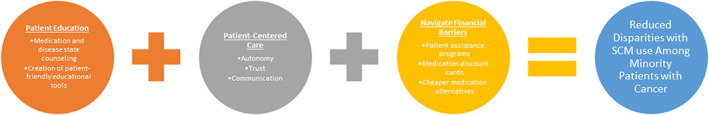
Potential framework for clinical pharmacists to reduce disparities in supportive care medication use in patients with cancer

Lastly, clinical pharmacists are uniquely positioned to be versed in the financial components of the cancer care process. Clinical pharmacists are aware of the difficulties patients face with medication access due to the high‐cost burden and can help patients navigate financial challenges. Clinical pharmacists can assist patients with selecting alternative medications that are covered by their insurance or direct patients to financial resources such as medication assistance programs or medication discount cards to minimize costs. Figure 2 provides a potential framework for clinical pharmacists to alleviate potential supportive care medication use disparities in patients with cancer.

## 5 CONCLUSION

Cancer guidelines recommend early palliative care integration into cancer treatment. Research has shown that palliative care not only improves the patient's QoL but also survival. But despite these findings, disparities still exist with the acquisition of supportive care services. Patients incur multiple barriers to improved medication use (eg, financial toxicity, and health literacy) and are often challenged to understand complex medication regimens. In collaboration with the multidisciplinary oncology team, clinical pharmacists can aid in the alleviation of supportive care medication use disparities. Clinical pharmacists add value to the care of cancer patients by utilizing a patient‐centered treatment and counseling approach that supports medication reconciliation, side‐effect mitigation, and financial risk minimalization to patients.

Despite the current knowledge about supportive care medication use, disparities in cancer treatment research gaps still exist. There needs to be a better understanding of the social and cultural factors that shape patients' perspectives toward utilizing supportive care medications. Research needs to be done to analyze the impact of insurance on the acquisition of supportive care medications. Most of the current studies have included only patients with Medicare, with little known about disparities with private insurance or younger patient populations.

## CONFLICT OF INTEREST

The authors declare no conflicts of interest.

## Supporting information


**Table S1.** PubMed database search strategy.
**Table S2.** Risk of bias assessment.
