# Supplementary material for: A scoping review on the relationship between race/ethnicity and the receipt of supportive care medications during cancer treatment: Implications for the clinical pharmacist
Source: J Am Coll Clin Pharm. 2022 Nov 13;5(12):1284–96. doi: 10.1002/jac5.1727 (PMC9957233; doi:10.1002/jac5.1727)
Supplement: Supplementary file 1 — Table S1. PubMed database search strategy. Table S2. Risk of bias assessment. [file JAC5-5-1284-s001.docx]

**Supplemental Table 1. PubMed Database Search Strategy**

| Search number | PubMed Search Query | Filters | Results |
| --- | --- | --- | --- |
| 11 | #6 AND #7 AND #8 AND #9 | English, from 2001/1/1 - 2021/12/31 | 106 |
| 10 | #6 AND #7 AND #8 AND #9 | English | 117 |
| 9 | "Cancer Pain"[Mesh] OR "cancer pain"[tiab] OR "malignant pain"[tiab] OR "Pain"[Mesh] OR "acute pain"[tiab] OR "deep pain"[tiab] OR "lightning pain"[tiab] OR "nocturnal pain"[tiab] OR "pain"[tiab] OR "pain response"[tiab] OR "pain syndrome"[tiab] OR "treatment related pain"[tiab] OR "chemotherapy induced nausea and vomiting"[tiab] OR "chemotherapy induced nausea and vomiting"[tiab] OR "chemotherapy-associated nausea and emesis"[tiab] OR "chemotherapy-associated nausea and vomiting"[tiab] OR "chemotherapy-induced nausea and emesis"[tiab] OR "chemotherapy-related nausea and emesis"[tiab] OR "chemotherapy-related nausea and vomiting"[tiab] OR "Vomiting"[Mesh] OR "Nausea"[Mesh] OR "nausea and emesis"[tiab] OR "nausea and vomiting"[tiab] OR "nausea"[tiab] OR "creatic nausea"[tiab] OR "nausea"[tiab] OR "nauseation"[tiab] OR vomit[tiab] OR vomiting[tiab] OR "Antiemetics" [Pharmacological Action] OR "Antiemetics"[Mesh] OR "anti emetic agent"[tiab] OR "antiemetic"[tiab] OR "antiemetic agent"[tiab] OR "antiemetic drug"[tiab] OR "antiemetics"[tiab] OR "Neurokinin-1 Receptor Antagonists" [Pharmacological Action] OR "Neurokinin-1 Receptor Antagonists"[Mesh] OR "nk1 antagonist"[tiab] OR "neurokinin 1 antagonist"[tiab] OR "neurokinin 1 receptor antagonist"[tiab] OR "neurokinin 1 receptor antagonists"[tiab] OR "neurokinin 1 receptor blocker"[tiab] OR "neurokinin 1 receptor blockers"[tiab] OR "neurokinin 1 receptor blocking agent"[tiab] OR "neurokinin-1 receptor antagonists"[tiab] OR "Antineoplastic Agents" [Pharmacological Action] OR "Antineoplastic Agents"[Mesh] OR "anti cancer drug"[tiab] OR "anti neoplastic agent"[tiab] OR "anticancer agent"[tiab] OR "anticancer drug"[tiab] OR "anticancerogen"[tiab] OR "anticarcinogen"[tiab] OR "anticarcinogenic agents"[tiab] OR "antineoplastic agent"[tiab] OR "antineoplastic agents"[tiab] OR "antineoplastic combined chemotherapy protocols"[tiab] OR "antineoplastic drug"[tiab] OR "antineoplastic peptide"[tiab] OR "antineoplastics"[tiab] OR "antitumor agent"[tiab] OR "antitumor drug"[tiab] OR "antitumour agent"[tiab] OR "antitumour drug"[tiab] OR "cancer chemotherapeutic agent"[tiab] OR "cancer inhibitor"[tiab] OR "carcinostatic drug"[tiab] OR "tumor inhibitor"[tiab] OR "tumour inhibitor"[tiab] | English | 2,030,693 |
| 8 | "Palliative Care"[Mesh] OR "supportive care"[tiab] OR "supportive care demand"[tiab] OR "supportive care need"[tiab] OR "supportive cancer care"[tiab] OR "palliation"[tiab] OR "palliative care"[tiab] OR "palliative consultation"[tiab] OR "palliative medicine"[tiab] OR "palliative radiotherapy"[tiab] OR "palliative surgery"[tiab] OR "palliative therapy"[tiab] OR "palliative treatment"[tiab] OR "symptomatic treatment"[tiab] OR "supportive care"[tiab] OR "hrql"[tiab] OR "health related quality of life"[tiab] OR "life quality"[tiab] OR "quality of life"[tiab] OR "supportive medication"[tiab] OR "Quality of Life"[Mesh] | English | 447,411 |
| 7 | "Neoplasms"[Mesh] OR "cancer"[tiab] OR "cancers"[tiab] OR "malignant neoplasia"[tiab] OR "malignant neoplasm"[tiab] OR "malignant neoplastic disease"[tiab] OR "malignant tumor"[tiab] OR "malignant tumour"[tiab] OR "neoplasia, malignant"[tiab] OR "tumor, malignant"[tiab] OR "tumour, malignant"[tiab] OR "Carcinoma"[Mesh] OR "carcinoma*"[tiab] OR "carcinoma 63"[tiab] OR "carcinoma, scirrhous"[tiab] OR "epithelial carcinoma"[tiab] OR "epithelial malignant tumor"[tiab] OR "epithelial malignant tumour"[tiab] OR "internal carcinoma"[tiab] OR "malignant epithelial tumor"[tiab] OR "malignant epithelial tumour"[tiab] OR "microcarcinoma"[tiab] OR "primary carcinoma"[tiab] OR "cancerology"[tiab] OR "clinical oncology"[tiab] OR "medical oncology"[tiab] OR "oncology"[tiab] OR "neoplasm*"[tiab] OR "acral tumor"[tiab] OR "acral tumour"[tiab] OR "embryonal and mixed neoplasms"[tiab] OR "germ cell and embryonal neoplasms"[tiab] OR "glandular and epithelial neoplasms"[tiab] OR "hormone-dependent neoplasms"[tiab] OR "neoplasia"[tiab] OR "neoplasm"[tiab] OR "neoplasms"[tiab] OR "neoplasms by histologic type"[tiab] OR "neoplastic disease"[tiab] OR "neoplastic entity"[tiab] OR "neoplastic mass"[tiab] OR "post-traumatic neoplasms"[tiab] OR "tumor"[tiab] OR "tumoral entity"[tiab] OR "tumoral mass"[tiab] OR "tumorous entity"[tiab] OR "tumorous mass"[tiab] OR "tumour"[tiab] OR "tumoural entity"[tiab] OR "tumoural mass"[tiab] OR "tumourous entity"[tiab] OR "tumourous mass"[tiab] OR tumor[tiab] OR tumour[tiab] OR "Neoplasm Metastasis"[Mesh] OR "cancer cell dissemination"[tiab] OR "cancer cell metastasis"[tiab] OR "cancer cell spread"[tiab] OR "cancer dissemination"[tiab] OR "cancer metastasis"[tiab] OR "cancer spread"[tiab] OR "carcinoma metastasis"[tiab] OR "disseminated tumor cell"[tiab] OR "disseminated tumour cell"[tiab] OR "metastases"[tiab] OR "metastasic type"[tiab] OR "metastasis"[tiab] OR "metastasis formation"[tiab] OR "metastatic cancer"[tiab] OR "metastatic cancers"[tiab] OR "metastatic carcinoma"[tiab] OR "metastatic carcinomas"[tiab] OR "metastatic disease"[tiab] OR "metastatic tumor"[tiab] OR "metastatic tumors"[tiab] OR "metastatic tumour"[tiab] OR "metastatic tumours"[tiab] OR "neoplasm metastasis"[tiab] OR "neoplastic cell dissemination"[tiab] OR "sarcoma metastasis"[tiab] OR "secondary cancer"[tiab] OR "secondary carcinoma"[tiab] OR "tumor dissemination"[tiab] OR "tumor metastasis"[tiab] OR "tumor migration"[tiab] OR "tumor spread"[tiab] OR "tumour dissemination"[tiab] OR "tumour metastasis"[tiab] OR "tumour migration"[tiab] OR "tumour spread"[tiab] OR metastasized[tiab] OR metastatic[tiab] | English | 4,016,605 |
| 6 | #1 OR #5 | English | 105,512 |
| 5 | #3 AND #4 | English | 105,512 |
| 4 | "Hispanic or Latino"[Mesh] OR "hispanic"[tiab] OR "hispanic americans"[tiab] OR "hispanic or latino"[tiab] OR "latino"[tiab] OR "latinx"[tiab] OR "latina"[tiab] OR latina*[tiab] OR "African Americans"[Mesh] OR "african american"[tiab] OR "african americans"[tiab] OR "afro-american"[tiab] OR "american negro"[tiab] OR "american blacks"[tiab] OR "black american"[tiab] OR "black person"[tiab] OR "african continental ancestry group"[tiab] OR "black man"[tiab] OR "black people"[tiab] OR "black person"[tiab] OR "black population"[tiab] OR "black race"[tiab] OR "black*"[tiab] OR "negro"[tiab] OR "negroes"[tiab] OR "negroid"[tiab] OR "negroid race"[tiab] OR "negroids"[tiab] OR "people of african ancestry"[tiab] OR "Indigenous Peoples"[Mesh] OR "aboriginal"[tiab] OR "aborigine"[tiab] OR "aborigines"[tiab] OR "indigenous people"[tiab] OR "indigenous peoples"[tiab] OR "native born"[tiab] OR "native people"[tiab] OR "natives"[tiab] OR "american indian"[tiab] OR "american indians or alaska natives"[tiab] OR "american native"[tiab] OR "american natives"[tiab] OR "american native continental ancestry group"[tiab] OR "amerindian"[tiab] OR "native american"[tiab] OR "central american indian"[tiab] OR "central american indians"[tiab] OR "indian, american"[tiab] OR "indians, central american"[tiab] OR "indians, north american"[tiab] OR "indians, south american"[tiab] OR "north american indian"[tiab] OR "north american indians"[tiab] OR "south american indian"[tiab] OR "south american indians"[tiab] OR "Asians"[Mesh] OR "asian"[tiab] OR "asians"[tiab] OR "biracial people"[tiab] OR "biracial person"[tiab] OR "biracial population"[tiab] OR "biracials"[tiab] OR "mixed-race people"[tiab] OR "multiracial people"[tiab] OR "multiracial person"[tiab] OR "multiracial population"[tiab] OR "multiracials"[tiab] OR "people of mixed race"[tiab] OR "person of mixed race"[tiab] OR "Racial Groups"[Mesh] OR "race"[tiab] OR "race factors"[tiab] OR "racial factor"[tiab] OR "racial factors"[tiab] OR "racial stocks"[tiab] OR racial[tiab] OR "ethnic or racial aspects"[tiab] OR "Ethnicity"[Mesh] OR "ethnicity"[tiab] OR ethnicities[tiab] OR "Whites"[Mesh] OR "caucasian"[tiab] OR "caucasian race"[tiab] OR "caucasoid"[tiab] OR "caucasoid race"[tiab] OR "caucasoids"[tiab] OR "european continental ancestry group"[tiab] OR "europeoid"[tiab] OR "europeoids"[tiab] OR "europid"[tiab] OR "europids"[tiab] OR "europoid"[tiab] OR "europoids"[tiab] OR "white people"[tiab] OR "white person"[tiab] OR "white population"[tiab] OR "white race"[tiab] OR "whites"[tiab] OR "people of european ancestry"[tiab] | English | 671,590 |
| 3 | "Health Disparity, Minority and Vulnerable Populations"[Mesh] OR "Healthcare Disparities"[Mesh] OR disparit*[tiab] OR inequalit*[tiab] OR inequit*[tiab] | English | 227,067 |
| 1 | "race disparity"[tiab] OR "race inequality"[tiab] OR "race inequity"[tiab] OR "racial disparity"[tiab] OR "racial inequality"[tiab] OR "racial inequity"[tiab] | English | 2,054 |

**Supplemental Table 2. Risk of Bias Assessment**

| Article | Citation # | Low Risk | Some Concerns | High Risk | Very High Risk |
| --- | --- | --- | --- | --- | --- |
| Booker et al. | 21 | x |  |  |  |
| Check et al. | 20 |  | x |  |  |
| Check et al. | 27 |  | x |  |  |
| Check et al. | 28 |  | x |  |  |
| Gomez et al. | 31 |  | x |  |  |
| Gurney et al. | 26 |  | x |  |  |
| Lamba et al. | 32 |  | x |  |  |
| Lu et al. | 22 |  | x |  |  |
| Pinheiro et al. | 27 |  | x |  |  |
| Wieder et al. | 23 |  | x |  |  |
| Hwang et al. | 33 |  |  | X  (Descriptive study) |  |
| Smith et al. | 24 |  |  |  | X  (Abstract) |
| Osazuwa-Peters et al. | 25 |  |  |  | X  (Abstract) |
| Dranitsaris et al. | 30 |  |  |  | X  (Abstract) |

**Interpretation of Results:**

**Low Risk:** There is the possibility of residual confounding that has not been controlled for (given the observational nature of the study), but otherwise little or no concern about bias in the result

**Some Concerns:** There is some concern about bias in the result, although it is not clear that there is an important risk of bias

**High Risk of Bias:** The study has some important problems: characteristics of the study give rise to a high risk of bias in the result

**Very High Risk of Bias:** The study is very problematic: characteristics of the study give rise to a very high risk of bias in the result
